# Thymic Epithelial Tumors phenotype relies on miR-145-5p epigenetic regulation

**DOI:** 10.1186/s12943-017-0655-2

**Published:** 2017-05-10

**Authors:** Teresa Bellissimo, Federica Ganci, Enzo Gallo, Andrea Sacconi, Claudia Tito, Luciana De Angelis, Claudio Pulito, Silvia Masciarelli, Daniele Diso, Marco Anile, Vincenzo Petrozza, Felice Giangaspero, Edoardo Pescarmona, Francesco Facciolo, Federico Venuta, Mirella Marino, Giovanni Blandino, Francesco Fazi

**Affiliations:** 1 Deptartment of Anatomical, Histological, Forensic & Orthopaedic Sciences, Section of Histology & Medical Embryology, Sapienza University of Rome, Rome, Italy; 2 Oncogenomic and Epigenetic Unit, “Regina Elena” National Cancer Institute, Rome, Italy; 3 Department of Pathology, “Regina Elena” National Cancer Institute, Rome, Italy; 4 Molecular Chemoprevention Unit, “Regina Elena” National Cancer Institute, Rome, Italy; 5 Department of Thoracic Surgery, Azienda Policlinico Umberto I, Sapienza University of Rome, Rome, Italy; 6 Pathology Unit, ICOT, Department of Medico-Surgical Sciences and Biotechnologies, Sapienza University of Rome, Latina, Italy; 7 Department of Radiological, Oncological, and Anatomo-pathological Science, Sapienza University of Rome, Rome, Italy and IRCCS Neuromed, Pozzilli, Italy; 8 Thoracic Surgery Unit, “Regina Elena” National Cancer Institute, Rome, Italy; 9 Fondazione Eleonora Lorillard Spencer Cenci, Rome, Italy

**Keywords:** microRNA, Thymic epithelial tumors, miR-145-5p, Thymoma, Thymic carcinoma, Epigenetic regulation

## Abstract

**Background:**

Thymoma and thymic carcinoma are the most frequent subtypes of thymic epithelial tumors (TETs). A relevant advance in TET management could derive from a deeper molecular characterization of these neoplasms. We previously identified a set of microRNA (miRNAs) differentially expressed in TETs and normal thymic tissues and among the most significantly deregulated we described the down-regulation of miR-145-5p in TET. Here we describe the mRNAs diversely regulated in TETs and analyze the correlation between these and the miRNAs previously identified, focusing in particular on miR-145-5p. Then, we examine the functional role of miR-145-5p in TETs and its epigenetic transcriptional regulation.

**Methods:**

mRNAs expression profiling of a cohort of fresh frozen TETs and normal tissues was performed by microarray analysis. MiR-145-5p role in TETs was evaluated in vitro, modulating its expression in a Thymic Carcinoma (TC1889) cell line. Epigenetic transcriptional regulation of miR-145-5p was examined by treating the TC1889 cell line with the HDAC inhibitor Valproic Acid (VPA).

**Results:**

Starting from the identification of a 69-gene signature of miR-145-5p putative target mRNAs, whose expression was inversely correlated to that of miR-145-5p, we followed the expression of some of them in vitro upon overexpression of miR-145-5p; we observed that this resulted in the down-regulation of the target genes, impacting on TETs cancerous phenotype. We also found that VPA treatment of TC1889 cells led to miR-145-5p up-regulation and concomitant down-regulation of miR-145-5p target genes and exhibited antitumor effects, as indicated by the induction of cell cycle arrest and by the reduction of cell viability, colony forming ability and migration capability. The importance of miR-145-5p up-regulation mediated by VPA is evidenced by the fact that hampering miR-145-5p activity by a LNA inhibitor reduced the impact of VPA treatment on cell viability and colony forming ability of TET cells. Finally, we observed that VPA was also able to enhance the response of TET cells to cisplatin and erlotinib.

**Conclusions:**

Altogether our results suggest that the epigenetic regulation of miR-145-5p expression, as well as the modulation of its functional targets, could be relevant players in tumor progression and treatment response in TETs.

**Electronic supplementary material:**

The online version of this article (doi:10.1186/s12943-017-0655-2) contains supplementary material, which is available to authorized users.

## Background

Thymic Epithelial Tumors (TETs) are rare tumors arising from the thymic epithelium and they show a propensity to recur and to metastasize that is difficult to predict [1]. Thymoma and thymic carcinoma (TC) are the most frequent TETs types [2]. Although TETs classification has been largely debated, now the World Health Organization (WHO) histologic classification is widely accepted and diffuse. WHO TETs subtypes are based on epithelial cell morphology and lymphocytic infiltration [3–5]. While type A, type AB and type B1 thymomas have a favorable prognosis, type B2 and especially type B3 thymomas and thymic carcinomas show an unfavorable outcome, in particular when diagnosed in an advanced tumor stage [6]. The 5-year recurrence rate reported by histotype was 4% for type A, 2%for type AB, 8% for type B1, 13% for type B2 and 14% for type B3, survival data being influenced by the fact that type A thymoma occur in older patients. Staging at diagnosis has been always considered the major prognostic factor [7, 8], whereas histotype classification has a limited prognostic relevance. Early diagnosis and complete surgical excision are the main factors contributing to a definitive treatment of TET. The unpredictable oncological properties of TETs, the preservation of the thymopoietic activity at variable degrees, and the frequent association with autoimmune diseases render TETs unique and difficult to manage [9]. Treatment strategies for newly diagnosed and recurrent TETs have evolved over time [10] and novel therapeutic strategies are emerging [11–14], even though the rarity of these tumors hindered the feasibility of large clinical trial slowing their development. Importantly, the oncogenic potential of these rare neoplasms is still largely undefined and a deeper molecular characterization could result in a relevant advance in their management greatly improving diagnosis, prognosis and treatment choice [15, 16]. In this direction, different studies investigated the epidermal growth factor receptor (EGFR) as a potential therapeutic target in TETs. Although EGFR mutations are rare in TETs, the receptor has been shown frequently overexpressed in thymomas and thymic carcinomas [17, 18]. However, the effectiveness of EGFR-targeting therapies in TETs has not been exhaustively clarified [12, 19–22]. Although single-case reports suggest that EGFR-tyrosine kinase inhibitors (Erlotinib or Gefitinib) or anti-EGFR mAb (Cetuximab) may be effective in some patients, two clinical phase II studies (44 patients in total) using Erlotinib and Gefitinib reported no complete remissions and only two partial responses [12, 15, 23]. These considerations suggest that studies addressing novel therapeutic strategies and the molecular basis of TETs are greatly needed. In this direction a recent work evidenced that Valproic Acid (VPA), a histone deacetilase (HDAC) inhibitor, might markedly increase the sensitivity of tyrosine kinase inhibitor (TKI)-resistant lung adenocarcinoma cells to Erlotinib [24]. Based on this evidence and on a recent phase I/II clinical trial highlighting that the combination treatment of Belinostat, an HDAC inhibitor, with chemotherapy agents was active and feasible in TETs [25], we focalized our attention to these epigenetic inhibitors as proof of concept to perform combination treatments in TET cells and to study the impact of the epigenetic regulation on TET phenotype.

Deregulation of epigenetic programs, as chromatin “histone code” at specific gene sites, and the expression of non-coding RNAs including microRNAs (miRNAs), cooperate with genetic alterations in the establishment and progression of tumors [26–28]. Interestingly, recent evidences suggest that miRNAs and epigenetic pathways appear to form a complex regulatory circuit that modulates the expression of an increasing number of genes in the genome. In particular, miRNAs can repress key enzymes that drive epigenetic remodeling [29]. Moreover, miRNAs can recruit Argonaute proteins and chromatin modifier enzymes by specific binding to complementary sites in gene promoters or by the interaction with target sites in nascent coding/non-coding transcript [30]. Although, epigenetics represents a novel field of research in TET biology, also these tumors are emerging as epigenetically driven cancer [31, 32]. Recently, specific genetic and epigenetic aberrations associated with different TET histotypes have been identified, providing insight into the genes involved in the pathogenesis of these tumors [33–42]. In several tumors miRNAs expression profiling is emerging as a significant tool for diagnosis, prognosis and treatment of cancer and recently, deregulation of miRNAs expression has been also established in TET patients [43, 44]. In particular, our group contributed to the identification of a signature of miRNAs deregulated in tumor versus normal tissues, some of which were also detected as circulating miRNAs in plasma [43, 45]. Interestingly, expression levels of specific onco-miRNAs, that we found up-regulated in the blood plasma collected from TET patients at surgery, resulted significantly reduced in follow-up samples [45]. Among the common miRNAs deregulated in all TET histotype groups in comparison with normal tissues, we observed the down-regulation of miR-145-5p in TETs, in line with what previously reported for other tumors [43, 46]. Several reports have revealed that the down-regulation of miR-145-5p expression is associated with neoplastic cell growth and proliferation as well as with cancer cell migration, invasion and metastasis, supporting miR-145-5p tumor suppressor activity in different tumor types [46–50]. The tumor suppressive functions of miR-145-5p were also reported in lung cancer and in malignant pleural mesothelioma (MPM), where the contribution of epigenetic transcriptional regulation to the abnormally low levels of miR-145-5p expression was recently described [51, 52]. Deciphering the molecular networks governed by miR-145-5p in TETs may be relevant for a deep characterization of these tumors and could lead to the design of new therapeutic strategies.

In this study we set out to address this question. We analyzed the mRNA expression profile in a fresh frozen cohort of tissues from TETs and normal counterparts, and selected the putative miR-145-5p targets among the mRNA differentially regulated. Thus we identified a 69-gene signature of miR-145-5p putative target mRNAs differently expressed between tumor and normal tissues whose expression was inversely correlated to that of miR-145-5p. We validated that Golm-1 and CDH-2, functionally involved in the control of cell motility [48, 50], are directly targeted by miR-145-5p also in TET cells. Genes controlling cell motility are indeed of particular interest in TET, as metastasis is a major issue in patients with advanced disease. Then we investigated the function of miR-145-5p in a TET cell line observing that miR-145-5p plays a tumor suppressor role also in this biological system as described in literature for others. Furthermore, we assessed that miR-145-5p expression is epigenetically regulated in this cell line. Indeed we evidenced that treatment of TET cells with epigenetic drugs resulted in the induction of miR-145-5p expression, with consequent down-regulation of its target genes. This resulted in antitumor effects in terms of cell cycle arrest and reduction of cell viability, colony forming ability and migration capability.

## Methods

### Sample collection

Two independent cohorts of fresh frozen specimens were utilized: four thymomas of different histotypes and two thymi derived from the Pathology Department at the Regina Elena National Cancer Institute; two thymomas and one thymus derived from the “Policlinico Umberto I” Hospital, Rome, Italy. The thymomas frozen samples included: one type A, two type AB, two type B2, one type B3. The normal thymi (normal thymic counterparts) included peritumoral thymi of the same frozen series. All the frozen specimens derived from extensive, prospective sampling of surgical specimens. Frozen primary tumor samples and matched normal thymi were checked for diagnosis on frozen section and for consistency with the final pathological diagnosis. Among the nineteen FFPE specimens used for IHC analysis, fifteen TET derived from the Pathology Department of Regina Elena National Cancer Institute, four TET derived from the “Policlinico Umberto I” Hospital, Rome, Italy [43]. There were five thymic carcinoma and fourteen thymomas of different histotypes (one Type A, five Type AB, three Type B1, three Type B2, two Type B3). Among the ten normal FFPE counterparts, eight peritumoral thymi derived from the Regina Elena National Cancer Institute, two from the “Policlinico Umberto I” Hospital, Rome, Italy. Normal peritumoral thymic tissues were selected from cases with a well-represented epithelial cell compartment similar to hyperplastic thymic tissue, or from involuted thymus with predominance of epithelial cells. Staining of normal peritumoral thymic tissues for Cytokeratin 19 (CK19) was used to evaluate the frequency of epithelial cells both in hyperplastic thymus and in involuted thymus [43]. Only primary tumors were included in the study. The patients were not treated with any radio or radio-chemotherapy before surgery. Independent Ethical Committees from collaborating Institutions have approved this study.

### RNA extraction, labeling and microarray hybridization

Fresh frozen samples were homogenized by gentleMACS dissociator (Miltenyi Biotec, Bologna, Italy) in 700 μl of Qiazol (Qiagen, Chatsworth, CA) and RNA was extracted using the miRNAeasy® kit (Qiagen, Chatsworth, CA) following the manufacturer’s instructions. The concentration purity and quality of total RNA were assessed using a Nanodrop TM 1000 spectrophotometer (Nanodrop Technologies, Wilmington, DE, USA) and the Agilent 2100 Bioanalyzer (Agilent, Santa Clara, California, USA). Total RNA for each specimen was labeled and used for microarray analysis of mRNAs expression on Affymetrix platform. Specifically, for mRNAs expression, 100 ng of total RNA from fresh frozen tissues was hybridized on “Affymetrix® Human Gene 2.0 ST Arrays 2.0” (Affymetrix, Santa Clara, California). Scanning and image analysis were performed using the Affymetrix GeneChip 3000 Scanner according to the Affymetrix GeneChip WT Terminal Labeling and Hybridization User Manual.

### Statistical analysis

Signals from gene expression profiling were background adjusted and quantile normalized. A permutation test and a false discovery procedure [53] were used to identify most deregulated genes between tumoral and normal samples. Genes presenting a *p*-value <0.05 for both *t*-test and permutation test were considered for further analyses. Principal component analysis and unsupervised hierarchical clustering were performed on deregulated features in order to discover groups of samples with different histotype. All signal processing analyses and statistical tests were performed by Matlab (The MathWorks Inc.). Several prediction target softwares were interrogated by using the web server tool MirWalk2 (http://zmf.umm.uni-heidelberg.de/apps/zmf/mirwalk2/) and the most predicted putative targets of a miRNA signature were selected to evaluate miRNA\mRNA positive and negative correlation. mRNAs predicted to be targeted by at least 2 prediction algorithms were considered. A Spearman correlation coefficient and relative *p*-value were obtained for each miRNA\mRNA pairs, and genes with *p*-values less than 0.05 were used for further analyses.

### Pathway prediction

Gene annotation enrichment analysis of the identified signatures of mRNAs deregulated in tumor *vs* normal samples was performed using DAVID program (https://david.ncifcrf.gov/) [54, 55].

### cDNA synthesis and RT-qPCR

Reverse Transcription and RT-qPCR quantification of miR-145-5p expression were performed respectively by TaqMan MicroRNA RT assay (Applied Biosystems, Foster City, CA, USA) and TaqMan MiRNA® Assays (Applied Biosystems, Foster City, CA, USA) according to the manufacturer's protocol. RNU6B and RNU49 were used as endogenous controls to standardize miR-145-5p expression in human samples. Whereas as endogenous control to standardize miR-145-5p expression in TC1889 cells was used the RNU19. All reactions were performed in duplicate. To validate data obtained by microarray RNA from 2 normal and 6 tumor samples used in array experiments was anlyzed by RT-qPCR for the expression of a subgroup of genes (Golm-1, Psat-1, CDH2). Reverse Transcription and qPCR﻿ were performed respectively by MMLV RT assay (Thermo Fisher Scientific, Waltham, MA USA) and Sybr green® assays (Applied Biosystems, Foster City, CA, USA) according to the manufacturer's protocol. GAPDH and RPL19 were used as endogenous controls to standardize gene expression. All reactions were performed in duplicate. Total RNA from TC1889 cells was extracted using the TRIZOL Reagents (Gibco® Thermo Fisher Scientific, Waltham, MA USA) and 500 ng of total RNA were reverse-transcribed at 37 °C for 60 min using High-Capacity RNA-to-cDNA Kit (Applied Biosystems, Foster City, CA, USA) and diluted 1:5 for the following PCR reactions. The list of primers used is showed in Additional file 1: Table S6.

### Immunohistochemistry

Golm-1 and CDH2 proteins expression were analyzed by immunohistochemistry (IHC) in a set of 19 FFPE thymic tumors and 10 normal counterparts from cases previously described and showing miR-145-5p deregulation [43]. 5 μm-thick sections were Haematoxylin and Eosin stained. Serial/subsequent sections were stained with the anti-Golm-1 antibody (#PA5-30622, Thermo Fisher Scientific, Waltham, MA USA) and the anti-CDH2 antibody (#ab18203, Abcam, Cambridge, UK) in the Ventana Staining System (Benchmark Ultra Ventana, Roche, Tucson, USA). Slide evaluation was independently and blinded performed by MM and EG. Overall inter-observer difference was 5%. In case of differing results, consensus was reached by joint evaluation. The following scoring approach in the assessment of Golm-1 and CDH2 immunostaining was used: score 0 = no staining or unspecific staining of tumor cells; score >1 from moderate to strong staining of more than 10% of tumor cells.

### Cell culture, transfection and treatment

Human Thymic Carcinoma cell line TC1889 was cultured in RPMI 1640 (Gibco® Thermo Fisher Scientific, Waltham, MA USA) containing 4.5 g/L glucose, 25 mM Hepes, 50 U/mL penicillin, 50 U/ml streptomycin and 10% heat-inactivated South-American Fetal Bovine Serum (FBS) (Gibco® Thermo Fisher Scientific, Waltham, MA USA) at 37 °C in incubator with humidified 5% CO2 atmosphere [40, 56].

Pre-miRNA-145 (#AM17100, Thermo Fisher Scientific, Waltham, MA USA) and Pre-miRNA Precursor Negative Control (#AM17110, Thermo Fisher Scientific, Waltham, MA USA), were transiently transfected at final concentration of 5nM using Lipofectamine RNAiMAX (Gibco® Thermo Fisher Scientific, Waltham, MA USA) according to the manufacturer’s instructions. For gene silencing experiments, TC1889 cells were transiently transfected with 250 pmols of Dicer-substrate short interfering RNAs (DsiRNAs) (IDT, Leuven, Belgium) for Golm-1, CDH2 and NC1 negative control for 72 h. For the inhibition of miR-145-5p expression, TC1889 cells were transiently transfected with hsa-miR-145-5p mirVana miRNA inhibitor (#MH11480, Thermo Fisher Scientific, Waltham, MA USA) and negative control #1 (#4464077, Thermo Fisher Scientific, Waltham, MA USA) at final concentration of 5nM using Lipofectamine RNAiMAX (Gibco® Thermo Fisher Scientific, Waltham, MA USA) according to the manufacturer’s instructions. TC1889 cells transfected for 48 h with the inhibitor of miR-145-5p or negative control were subsequently treated with 0.5 mM Valproic acid sodium salt (VPA) (#P4543, Sigma-Aldrich, Darmstadt, Germany) for additional 24 h. Treatment with VPA (#P4543, Sigma-Aldrich, Darmstadt, Germany) as single agent was performed at a concentration of 3 mM (or as indicated) for the indicated time. For the combination treatment TC1889 cells were treated for 8 h with VPA (3 mM) and subsequently with or without Cisplatin (CDDP, 7.5μg/mL) (Pfizer, New York, NY, USA), for additional 48 h.

Erlotinib (#5083 Cell Signaling, Danvers, MA, USA) was used in TC1889 cells as single agent in a dose-dependent treatment (as indicated). In Fig. 6d Erlotinib was used at 2.5 μM and 5 μM as single agent or in co-treatment with VPA (3 mM) for 72 h.

### Lysate preparation and immunoblotting analysis

Cells were lysed in 2% SDS buffer (25 mM Tris–HCl pH 7.5, 100 mM Nacl, 3 mM EDTA, 7% Glycerol) and fresh protease inhibitors. Extracts were sonicated for 10 s and centrifuged at 12000 × rpm for 10 min to remove cell debris. Western blotting was performed using the following primary antibodies: mouse monoclonal anti-Gapdh 6C5 (#sc32233, Santa Cruz Biotechnology, Heidelberg Germany); mouse monoclonal anti-b-Actin (ACTBD11B7) (#sc-81178, Santa Cruz Biotechnology, Heidelberg Germany); mouse monoclonal anti-Tubulin B512 (#T5168, Sigma-Aldrich, Darmstadt, Germany); rabbit polyclonal anti-Golm1 (#PA5-30622, Thermo Fisher Scientific, Waltham, MA USA); rabbit monoclonal anti-Synaptophysin (#ab32127, Abcam, Cambridge, UK); mouse polyclonal anti-Psat1 (#PA5-22124, Thermo Fisher Scientific, Waltham, MA USA), rabbit polyclonal anti-CDH2 (#ab18203, Abcam, Cambridge, UK), rabbit monoclonal anti-EGFR (#C74B9, Cell Signaling, Danvers, MA, USA) and rabbit polyclonal anti-p21 (12D1) (#2947 Cell Signaling, Danvers, MA, USA). As secondary antibodies goat anti-mouse and anti-rabbit conjugated to horseradish peroxidase were used (Bethyl Laboratories, Montgomery, TX, USA). Western blot analysis was performed with the aid of the enhanced chemiluminescence system (Thermo Fisher Scientific, Waltham, MA USA). ECL detection was done using a ChemiDoc-It Imaging System (UVP, Upland, CA) instrument.

### Transwell migration assay

Migration assay was performed by using a 24-well plate with a non-coated 8-mm pore size filter in the insert chamber (BD Falcon, Franklin Lakes, NJ, USA). Cells were transfected with pre-miRNA145 (for 120 h), or silenced with siGolm-1 at 250 pmoli (for 72 h), or silenced with siCHD2 at 250 pmoli (for 72 h) or treated with 3 mM VPA (for 72 h). After transfection or treatment, 5×10^4^ cells were resuspended in TC1889 medium without FBS and seeded into the insert chamber. Cells were allowed to migrate for 24 h into the bottom chamber containing 0,7 ml of TC1889 medium containing 30 ng/ml of epithelial growth factor (EGF) (#E9644, Sigma-Aldrich, Darmstadt, Germany) in a humidified incubator at 37 °C in 5% CO2. Cell migration was measured by counting the number of cells stained with DAPI on the underside of the membrane.

### Clonogenic assays

TC1889 cell lines were grown to 70% confluence and treated with VPA 3 mM. After 72 h of treatment, cells were detached and seeded at 1×10^3^ cells/well into 6-well dishes in drug-free media. Fresh media (25%) was added every three days. Colonies were stained with crystal violet and were counted after 10–14 days by ImageJ software.

### Immunocytochemistry and morphological analysis

For immunocytochemistry assay cells were seeded onto glass coverslips at 25×10^4^ cells/well into 6-well dishes and transfected with pre-miRNA-145 (#AM17100, Thermo Fisher Scientific, Waltham, MA USA) and pre-miRNA Precursor Negative Control (#AM17110, Thermo Fisher Scientific, Waltham, MA USA), at final concentration of 5nM for 96 h or treated with VPA for 72 h. Cells were then fixed with 4% formaldehyde in PBS for 15 min at room temperature (RT). Cells were permeabilized with 1% Triton X-100 in PBS for 10 min. After washing with PBS, the coverslips were incubated with the indicated antibody diluted in 5% BSA/PBS for 1 h at RT. Cells were incubated with peroxidase inhibitor before primary antibody incubation. Protein staining was revealed through DAB enzymatic reaction while nuclei were counterstained with hematoxylin. Morphological analysis was performed by the Wright-Giemsa-staining according to the manufacturer’s instructions.

### Cell cycle analysis

For cell cycle analysis, 2×10^5^ cells were resuspended in 50% FCS, fixed in 70% ethanol for 24 h, incubated with 50 μg/ml propidium iodide (Sigma-Aldrich, Darmstadt, Germany) and 50 units/ml Dnase-free RNase A (Sigma-Aldrich, Darmstadt, Germany) and analyzed after 3 h (1×10^4^ events) using an Epics XL Cytometer (Beckman Coulter, Brea, CA, USA).

### ATPlite Luminescence Assay System

ATPlite™ Luminescence Assay was performed by using a 96-well plate with 2×10^4^ cells/well and cell viability was evaluated following the manufacturer’s instructions. Luminescence was read by the EnSpire® Multimode Plate Reader (PerkinElmer, Whaltman, MA, USA).

### Formaldehyde cross-linking and chromatin immunoprecipitation

Formaldehyde cross-linking and chromatin immunoprecipitation were performed as described [57]. The chromatin solution was immunoprecipitated with an anti-acetyl-Histone H4 antibody (#06-866 Millipore, Billerica, MA, USA) or no antibody as negative control. Cyclin B1 first intron was amplified as negative control. Primers for the qPCR amplification of different miR-145-5p upstream genomic regions and negative control are reported in Additional file 1: Table S6.

### Plasmid construction and dual-luciferase reporter assay

The luciferase reporter assay was performed by using vectors kindly provided us by Dr Naohiko Seki for Golm-1 [50] and Dr Peng Gao for CDH2 [48]. Briefly as described, for Golm-1 the partial wild-type sequences of the Golm-1 3’-UTR or the mutant derivative lacking the miR-145-5p target sites (positions 260–267 of the Golm-1 3’-UTR) were inserted between the XhoI–PmeI restriction sites in the 3’UTR of the hRluc gene in the psiCHECK-2 vector (C8021; Promega, Madison, WI, USA) [50]; while for CDH2 the 3’-UTR sequence of CDH2 carrying a putative miR-145-5p binding site was amplified by PCR and cloned into a Taq-amplified (TA) vector (pTA2, Takara, Ostu, Shiga, Japan) and then subcloned into a pmirGLO miRNA target expression vector (Promega, San Lius Obispo, CA, USA) [48]. The TC1889 cells were transfected for 48 h with 50 ng of the vectors and pre-miRNA-145 (#AM17100, Thermo Fisher Scientific, Waltham, MA USA) or pre-miRNA Precursor Negative Control (#AM17110, Thermo Fisher Scientific, Waltham, MA USA) as indicated, using Lipofectamine RNAiMAX (Gibco® Thermo Fisher Scientific, Waltham, MA USA) according to the manufacturer’s instructions. The activities of firefly and renilla luciferases in cell lysates were determined with a dual-luciferase assay system (E1910; Promega, Madison, WI, USA). Normalized data for Golm-1 and CDH2 were calculated, respectively, as the ratio of renilla/firefly and firefly/renilla luciferase activities. The experiments were performed in duplicates.

## Results

### Differential mRNA expression between thymic epithelial tumors and normal thymic tissues

**Fig. 1:**
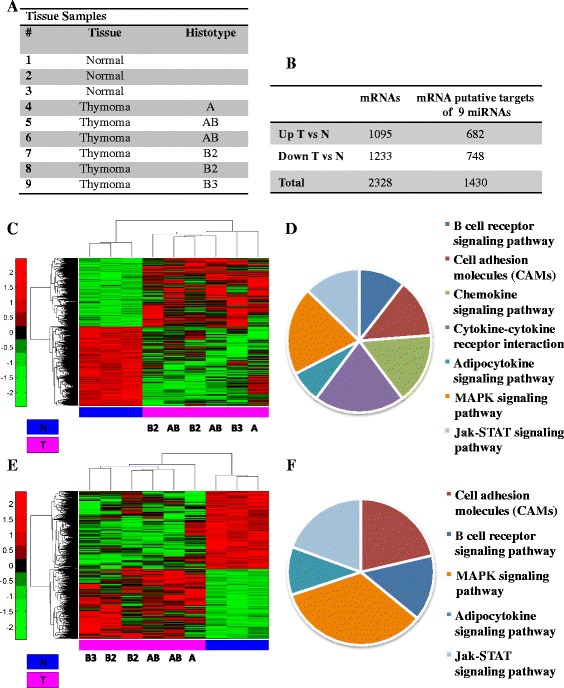
Gene expression profiling of Thymoma versus normal Thymus tissues. **a** Table representing the normal and tumor samples used in the experiment from two different cohorts and analyzed for gene expression profile. **b** Table showing the number of genes up and down-regulated in tumor versus normal tissues. **c** Unsupervised clustering analysis representing the 2328 genes differentially expressed between tumor and normal thymus tissues (*p*-value < 0.05, see material and methods). **d** Pie chart representing pathway enrichment analysis of the deregulated genes by David tools (*p*-value < 0.05). **e** Unsupervised clustering analysis representing the putative targets of the best 9 of 22 miRNAs common to histotype groups of thymic tumors compared to normal tissue (see material and methods and Ganci et al., [43]) **f**) Pie chart representing pathway enrichment analysis of the deregulated genes by David tools (*p*-value < 0.05)

Starting from a selection of frozen tissue samples showing high RNA quality, we performed a gene expression profiling using 6 TET specimens and 3 normal counterparts (Fig. 1a). We identified 2328 genes differently expressed between tumoral (T) and normal (N) samples. Among these genes 1095 were up-regulated and 1233 were down-regulated in thymic tumors (Fig. 1b and c and Additional file 2: Table S1). By unsupervised hierarchical clustering and Principal component analysis (PCA) we assessed that they were able to clearly separate tumor tissues from normal samples (Fig. 1c and Additional file 3: Figure S1A). Functional analysis of these genes evidenced that many of them converge on common pathways. In particular we identified Cell adhesion molecules pathway, B cell receptors signaling pathways, and additional pathways related to cancer phenotype such as MAPK and Jak-Stat signaling pathways (Fig. 1d and Additional file 4: Table S2).

We next searched whether putative targets of the miRNAs previously identified as commonly deregulated in all TET histotype groups in our previous work were present in the list of deregulated mRNAs [43]. Specifically, we considered the 9 miRNAs most strongly deregulated in TET (5 up-regulated: hsa-miR-34c-5p, hsa-miR-141-3p, hsa-miR-205-3p, hsa-miR-455-5p, hsa-miR-132-3p, and 4 down-regulated: hsa-miR-486-5p, hsa-miR-630, hsa-miR-451a, hsa-miR-145-5p). By using prediction algorithms on the list of genes deregulated between tumor and normal samples we identified a number of mRNAs, which are putatively targeted by at least one of the nine deregulated miRNAs. Evaluation of the Spearman correlation coefficient between the mRNA/miRNA pairs led to the identification of 1430 putative targets genes with expression pattern significantly correlated to the miRNAs (Fig. 1b and e and Additional file 3: Figure S1B); among these genes, 682 were up-regulated and 748 down-regulated in thymic tumors (Fig. 1b and e and Additional file 5: Table S3). Functional classification analysis identified for these genes similar pathways as those described above, except for Chemokine and Cytokine-cytokine receptors signaling pathways (Fig. 1f and Additional file 6: Table S4).

### Identification of miR-145-5p inversely correlated target mRNAs in TET tumors

**Fig. 2:**
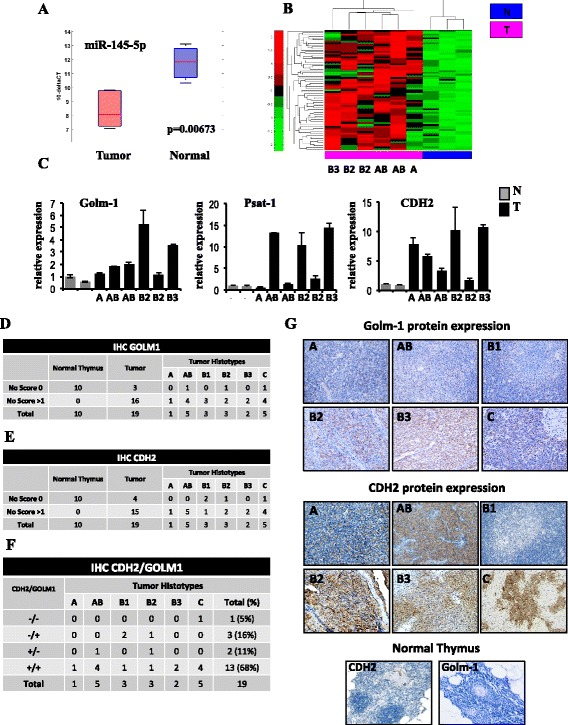
Expression profiling of putative targets of miR-145-5p in Thymoma versus normal Thymus tissues. **a** Box plot showing the modulation of miR-145-5p expression in the group of 6 tumors and 3 normal samples used to evaluate gene expression profiling. **b** Unsupervised clustering analysis representing the expression of putative targets of miR-145-5p inversely correlated to miR-145-5p expression in thymic tumors compared to normal tissues. **c** Validation by RT-qPCR of mRNA expression of three putative targets of miR-145-5p, Golm-1, Psat-1 and CDH2, in the same dataset of samples used for gene expression profiling (2 normal tissues and 6 tumors). **d**-**e** Table showing the number of Golm-1 and CDH2 positive (score > 1) and negative (score 0) samples in the subset of normal and tumor tissues of each histotype. **f** Table showing the number of Golm-1 and CDH2 co-expressing samples in the subset of tumor tissues of each histotype. **g** Expression of Golm-1 and CDH2 protein by immunohistochemistry (IHC) analysis in a representative normal thymus and tumor tissues of each histotype

Among the best 9 miRNAs deregulated in all histotype groups of thymic tumors we previously evidenced the down-regulation of miR-145-5p [43]. miR-145-5p down-regulation is emerging as a common feature in various malignancies [46–50]. Down-regulation of miR-145-5p expression has been also confirmed in this selection of frozen tissue samples (Fig. 2a). Analysis of miR-145-5p putative targets evidenced a 69-gene signature of miR-145-5p putative target mRNAs whose expression is inversely correlated to that of miR-145-5p (Spearman correlation coefficient and relative *p*-value < 0.05) (Fig. 2b and Additional file 7: Table S5). Principal component analysis (PCA) and unsupervised hierarchical clustering of these 69 miR-145-5p putative targets show that these genes not only separate tumor from normal tissues but also, interestingly, clearly distinguish clinically relevant histotype groups (A/AB from B2/B3) (Additional file 3: Figure S1C). Validation of selected mRNAs was performed on the same patients cohort by RT-qPCR and, according to the expression profiling results, we evidenced the up-regulation of Golm-1, Psat-1 and CDH2 in tumor tissues compared to normal samples (Fig. 2c). The protein up-regulation of Golm-1 and CDH2 was also assessed by immunohistochemistry (IHC) using a series of formalin-fixed paraffin embedded (FFPE) tissues from 19 TET of different histotype (one Type A, five Type AB, three Type B1, three Type B2, two Type B3 and five Thymic Carcinoma) and ten normal counterparts included in our previous work [43]. As summarized in the Fig. 2d-f and representatively reported in the Fig. 2 g the IHC showed that both Golm-1 and CDH2 proteins are expressed at higher levels (arbitrary score >1) in tumors than in normal samples. Interestingly, 68% of tumor tissues showed co-expression of Golm-1 and CDH2 (Fig. 2f). Golm-1 and CDH2 proteins resulted not expressed or expressed at low levels (arbitrary score =0) in all the normal tissues analyzed (Fig. 2d, e and g).

By using luciferase reporter assay we validated that CDH2 and Golm-1 are miR-145-5p target genes also in the Thymic Carcinoma cell lines TC1889, as already published, respectively, for gastric [48] and prostate cancer [50]. The vector containing wild-type fragment of the 3’-UTR of Golm-1 mRNA or the mutant derivative lacking the putative miR-145-5p recognition sequence (position 260–267 of the Golm-1 3’UTR) [50] was transfected together with miR-145-5p or negative control in TC1889 cells. Luciferase expression of the wild-type construct was significantly reduced when miR-145-5p was expressed with respect to the negative control (Additional file 8: Figure S2A). Repression was abolished when the mutant miR-145-5p derivative was utilized (Additional file 8: Figure S2A). Similarly, the vector containing the sequence of the 3’-UTR of CDH2 mRNA carrying a putative miR-145-5p binding site [48] was transfected together with miR-145-5p or negative control in TC1889 cells. Luciferase expression was significantly reduced when miR-145-5p was expressed with respect to the negative control (Additional file 8: Figure S2B). Repression was not observed when the empty vector was utilized (Additional file 8: Figure S2B).

These data suggested that miR-145-5p bound directly to specific sites in the 3’UTR of Golm-1 and CDH2 mRNAs.

**Fig. 3:**
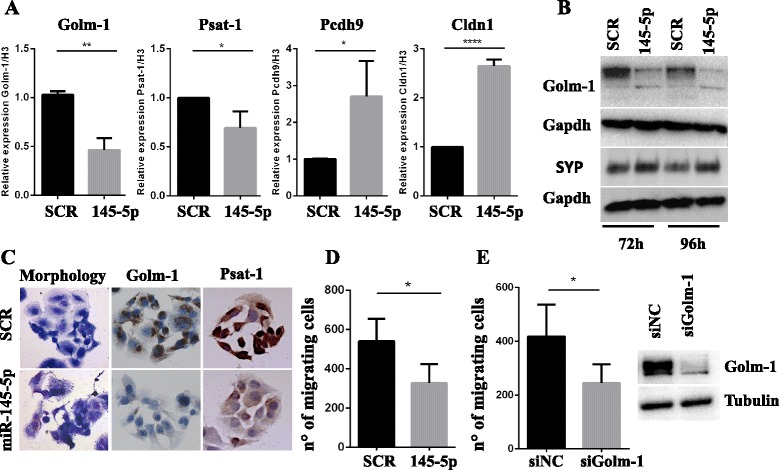
Overexpression of miR-145-5p in a Thymic Carcinoma (TC1889) cell line.** a** RT-qPCR to evaluate the expression levels of miR-145-5p inversely correlated (Golm-1 and Psat-1) and positively correlated (Pcdh9 and Cldn1) genes after miR-145-5p overexpression in TC1889 cells. **b** Western-blot analysis of Golm-1 and Synaptophysin (SYP) proteins after 72 h and 96 h of miR-145-5p overexpression in TC1889 cells. **c** Morphological and IHC analyses for Golm-1 and Psat-1 of miR-145-5p overexpression in TC1889 cells after 144 h or 96 h respectively. **d**-**e** Evaluation of the impact of miR-145-5p overexpression or siRNA for Golm-1 (siGolm-1) in TC1889 by migration assay after 120 h and 72 h respectively. P-value was calculated by unpaired *t*-test and a value of *P* ≤ 0.05(*), *P* ≤ 0.01(**) and *P* ≤ 0.0001(****) was considered statistically significant

We next evaluated the expression levels of miR-145-5p putative target mRNAs after overexpression of miR-145-5p in TC1889 cell line. As expected, we observed that the miR-145-5p overexpression resulted in the down-regulation of Golm-1 and Psat-1 (negatively correlated to the expression of miR-145-5p in the microarray analysis of TET samples) and in the up-regulation of Pcdh9 and Cldn1 (positively correlated to the expression of miR-145-5p in the microarray analysis of TET samples) (Fig. 3a and Additional file 8: Figure S2C). Accordingly, miR-145-5p overexpression resulted in the protein level down-regulation of Golm-1 and Psat-1, as assessed by Western-blot and immunocytochemistry (Fig. 3b and c). Interestingly, the overexpression of miR-145-5p, as well as the silencing of its targets Golm-1 (siGolm-1) and CDH2 (siCDH2) by siRNA, also led to reduced tumorigenic features of these cells, as evidenced by their reduced migration capability (Fig. 3d and e and Additional file 8: Figure S2D), supporting their contribution to TET phenotype. In addition, miR-145-5p overexpression led to morphological changes with increased cell-cell contacts and appearance of cells with a neuroepithelial-like features whose existence has been previously reported in Thymic cultures [58] (Fig. 3c). This agrees with the increased levels of the synaptophysin protein, a neuroendocrine differentiation marker, observed after miR-145-5p overexpression (Fig. 3b).

### Inhibition of HDAC activity releases miR-145-5p expression and reduces tumor phenotype of TET cells

**Fig. 4:**
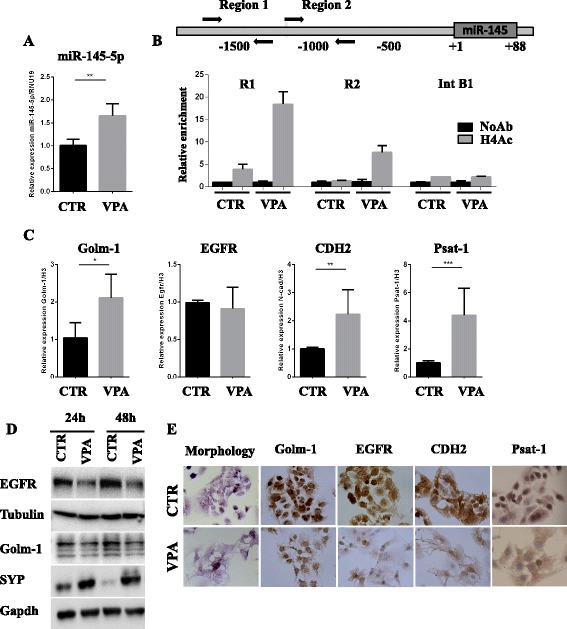
Inhibition of HDAC activity releases miR-145-5p expression resulting in miR-145-5p target genes modulation. **a** RT-qPCR to evaluate the expression levels of miR-145-5p after 72 h of 3 mM VPA treatment of TC1889 cells. **b** Schematic representation miR-145-5p upstream genomic region (upper panel) and Chromatin Immunoprecipitation (ChIP) analysis (lower panel) to evaluate the histone H4 acetylation (H4Ac) status of R1 and R2 miR-145 upstream genomic regions (R1 located at -1500 bp and R2 located at -1000pb) after 24 h of 3 mM VPA treatment in TC1886 cells. The intronic region of CCNB1 gene (Int B1) was used as negative control. **c** RT-qPCR to evaluate the expression levels of Golm-1, EGFR, CDH2 and Psat-1 after 72 h of 3 mM VPA treatment of TC1889 cells. **d** Wester blot analysis to evaluate Golm-1, EGFR and SYP after 24 h and 48 h of 3 mM VPA treatment of TC1889 cells. **e** Morphological and IHC analyses for Golm-1, EGFR, CDH2 and Psat-1 after 72 h of 3 mM VPA treatment of TC1889 cells. *P*-value was calculated by unpaired *t*-test and a value of *P* ≤ 0.05(*), *P* ≤ 0.01(**) and *P* ≤ 0.001(***) was considered statistically significant

**Fig. 5:**
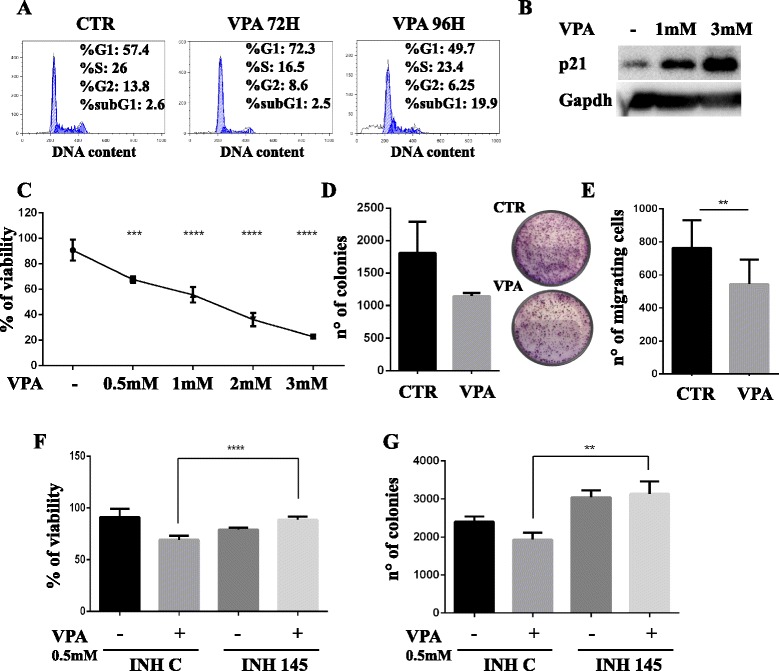
Inhibition of HDAC activity reduces tumor characteristics of TET cells. **a** Representative cell cycle analysis of TC1889 cells after 72 h and 96 h of 3 mM VPA treatment. **b** Western-blot analysis to evaluate the expression levels of p21 after 72 h of 1 mM or 3 mM VPA treatment. **c** ATPlite analysis to evaluate the viability of TC1889 cell after 24 h of a dose-dependent VPA treatment. **d** Evaluation of the impact of 3 mM VPA treatment for 72 h on TC1889 colony forming capability. The experiment was performed in triplicate and the graphical plot of the average (left panel) and a representative image (right panel) were reported. **e** Evaluation of the impact of 3 mM VPA treatment for 72 h on TC1889 migration capability. The experiment was performed in triplicate. **f**-**g** ATPlite analysis **f** and colony forming capability (**g**) of TC1889 cells treated for 48 h with the inhibitor of miR-145-5p (INH 145) or negative control (INH C) and subsequently treated with VPA 0.5 mM for additional 24 h. The experiment was performed in triplicate. *P*-value was calculated by unpaired *t*-test and a value of *P* ≤ 0.01(**), *P* ≤ 0.001(***) and *P* ≤ 0.0001(****) was considered statistically significant

To verify whether miR-145-5p expression is regulated at epigenetic level in Thymic Carcinoma (TC1889) cells, we evaluated the effect of an HDAC inhibitor (Valproic Acid, VPA) on miR-145-5p expression. As shown in Fig. 4a, miR-145-5p is significantly up-regulated following VPA treatment (72 h). According to this result, we evidenced, by Chromatin Immunoprecipitation (ChIP) analysis, an increased histone H4 acetylation (H4Ac) level following 3 mM VPA treatment (24 h) on miR-145 upstream genomic regions R1 and R2 (R1 located at -1500 bp and R2 located at -1000 bp) (Fig. 4b). This increase was not present on a negative control genomic region (intronic region of CCNB1 gene, indicated as Int B1). Interestingly, we observed that, although the VPA treatment significantly up-regulates the mRNA expression levels of Golm-1, Psat-1 and CDH2 (Fig. 4c), the protein expression of these miR-145-5p targets was down-regulated after VPA treatment, as assessed by WB and immunocytochemistry, suggesting that miR-145-5p inhibitory activity on translation of these targets is maintained and prevalent (Fig. 4d and e). Moreover, we evidenced that VPA treatment increases the expression levels of the synaptophysin protein and induces morphological changes as previously described in the miR-145-5p overexpressing cells (Fig. 4d and e and Fig. 3b-c). To further characterize the impact of VPA on TET phenotype of TC1889 cells, we evaluated its effect on cell cycle distribution. We observed that VPA treatment induced G1 arrest after 72 h, cell death after 96 h (Fig. 5a) and a dose-dependent induction of cell cycle master regulator p21 after 72 h of treatment (Fig. 5b). In addition, VPA treatment (24 h) inhibited cell viability in a dose dependent manner (Fig. 5c). Finally, as shown in Fig. 5d and e, we evidenced that VPA treatment (72 h) inhibited the colony forming ability and migration capability of TC1889 cells. Of note, to support that the impact of VPA on TET phenotype was, at least in part, mediated by the induction of miR-145-5p, we evaluated the cell viability and the colony forming capability of VPA-treated TC1889 cells after the inhibition of miR-145-5p expression. We observed that the treatment of TC1889 cells with the miR-145-5p inhibitor (INH-145) significantly impaired the VPA-dependent reduction of cell viability and colony forming capability compared to the same cells treated with a scramble inhibitor (INH-C) (Fig. 5f and g, Additional file 8: Figure S2E).

### Inhibition of HDAC activity sensitizes TET cells to chemotherapy and to EGFR tyrosine kinase inhibitor

**Fig. 6:**
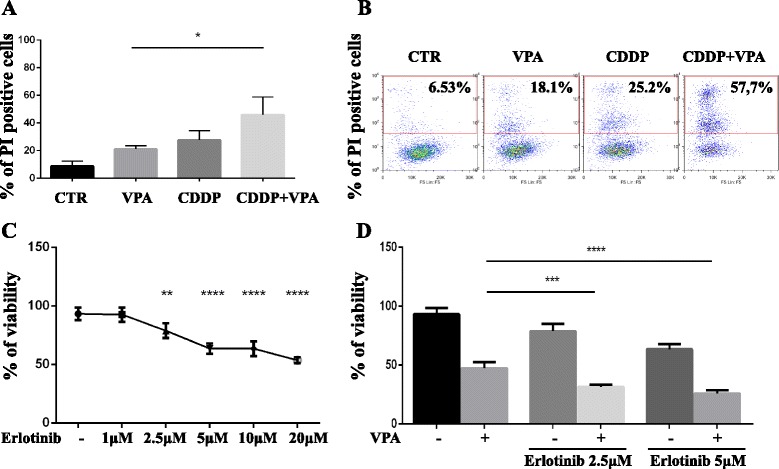
Inhibition of HDAC activity sensitizes TET cells to chemotherapy. **a**-**b** FACS analysis to evaluate the percentage of TC1889 Propidium Iodate (PI) positive cells treated with Cisplatin (CDDP, 7.5μg/mL) for 48 h and pretreated or not with 3 mM of VPA for 8 h as indicated. The experiment was performed in triplicate and the graphical plot of the average (**a**) and a representative FACS analysis (**b**) were reported. **c** ATPlite analysis to evaluate the viability of TC1889 cells after 72 h of a dose-dependent treatment with Erlotinib. **d** ATPlite analysis in TC1889 cells co-treated with Erlotinib (2.5μM or 5μM) and 3 mM of VPA for 72 h. The experiment was performed in triplicate. *P*-value was calculated by unpaired *t*-test and a value of P ≤ 0.05(*), *P* ≤ 0.01(**), *P* ≤ 0.001(***) and *P* ≤ 0.0001(****) was considered statistically significant

To evaluate whether VPA treatment modulates the sensitivity of TET cells to chemotherapeutic treatment, we pretreated Thymic Carcinoma cells for 8 h with VPA (3 mM) and subsequently we added or not Cisplatin (CDDP, 7.5μg/mL) for additional 48 h. FACS analysis evidenced that VPA treatment sensitizes TET cells to CDDP-dependent cell death (Fig. 6a and b).

We next evaluated the effect of combined therapy of VPA plus Erlotinib, an EGFR tyrosine kinase inhibitor, being Erlotinib under investigation as potential therapeutic agent in TET, especially in patients with heavily pretreated thymoma. EGFR protein is indeed highly expressed in TET and also in TC1889 cells (as previously shown in Fig. 4d), where its level is decreased following VPA treatment.

As shown in Fig. 6c, dose-dependent treatment with Erlotinib for 72 h significantly affects the viability of TC1889 cells. To evaluate whether VPA treatment modulates the sensitivity of TET cells to Erlotinib we co-treated TET cells with VPA (3 mM) and Erlotinib at 2.5 μM and 5 μM. As shown in Fig. 6d viability of TC1889 cells is significantly reduced after treatment with VPA + Erlotinib compared to single treatments.

## Discussion

Epigenetic therapy is recognized as an effective and well-tolerated treatment of cancer [59]. In particular, histone deacetylases (HDACs) are considered to be among the most promising targets in drug development for cancer therapy [60, 61].

To translate in TET management the therapeutic experience on epigenetic drugs already acquired in other tumors [59, 62], we explored here the possibility that Valproic Acid (VPA), an HDAC inhibitor, might synergize with other drugs to impair the epigenetic mechanisms at the basis of TET phenotype. To date chemotherapy or targeted therapy represent the frontline for the medical treatment of TETs although the rarity of this tumor type has precluded it from large phase II and III clinical trial investigations, and new drug development for TET treatment has progressed slowly [12].

Systemic chemotherapy is used for induction therapy, in cases of stage III/IVA, and currently represents the standard therapy for metastatic or inoperable refractory/recurrent disease [10]. Cisplatin-based combined chemotherapy currently represents the standard therapy for TETs with an important contribution of anthracyclines [12]. Cisplatin-based regimens, such as the PAC scheme (cisplatin, adriamycin, and cyclophosphamide) are most frequently used [63] and recently the use of belinostat, a histone deacetylase (HDAC) inhibitor, administered before and in combination with PAC, indicated that the combination is active in TETs [25].

Also targeted therapy has recently emerged in TET management and the use of EGFR-tyrosine kinase inhibitors (TKI) (Erlotinib or Gefitinib) or anti-EGFR mAb (Cetuximab) are currently under investigation as potential therapeutic agents in TET, especially in patients with heavily pretreated thymoma [12, 23, 64–66]. Interestingly, a recent work highlighted that the histone deacetylase (HDAC) inhibitor Valproic Acid (VPA) might markedly increase the sensitivity of TKI-resistant lung adenocarcinoma cells to Erlotinib [24].

We here demonstrate that the inhibition of HDAC activity by VPA sensitizes TET cells to Cisplatin (CDDP) or to EGFR tyrosine kinase inhibitor, supporting the possibility of a combination approach to improve the therapeutic response of TET malignancies. Of note, the treatment of VPA as single agent is able itself to impact on TET phenotype as evidenced by the induction of cell cycle arrest and reduction of cell viability, colony forming ability and migration capability in TET cells. These results support the involvement of epigenetic mechanisms in TETs. Specifically, VPA treatment is able to induce chromatin-remodeling events on the upstream regulatory regions of miR-145-5p, resulting in the induction of miR-145-5p expression and supporting its epigenetic de-regulation in TET cells. Following VPA treatment, miR-145-5p shows not only increased expression levels, but also the ability to exert its activities in TC cells. Indeed, while following VPA treatment we observe increased mRNA level of miR-145-5p target genes Golm-1, CDH2 and Psat-1, which is probably the effect of a general increase in transcription rate due to HDAC inhibition, protein analysis of these target genes revealed a clear decrease in their expression level, indicating the activation of a strong post-transcriptional control program on these mRNAs. These results suggest that the mechanisms controlling the translation of these mRNA are predominant on the transcriptional ones during VPA treatment.

Contrarily to Golm-1, CDH2 and Psat-1, no changes in EGFR mRNA level following VPA treatment were observed, while protein analysis of this miR-145-5p target gene revealed a clear decrease in its expression level, indicating the activation of a strong post-transcriptional control program on this mRNA. These results suggest that also for this miR-145-5p target gene the mechanisms controlling its translation are predominant on the transcriptional ones during VPA treatment in TET cells. Moreover, it was recently reported the involvement of miR-145-5p in the negative regulation of EGFR expressions at both mRNA and protein levels [67]. Thus, we cannot exclude the involvement of miR-145-5p in the transcriptional control of EGFR regulation also in TET cells.

The increased miR-145-5p activity also contributes, even if not the solely responsible, to the functional impact of VPA on TET phenotype, evaluated as cell viability and colony forming capability. In this context, the identification of a signature of genes positively and negatively associated to miR-145-5p expression could strongly contribute to the deciphering of the molecular mechanisms governed by or governing miR-145-5p expression. In this direction we have identified a 69-gene signature of miR-145-5p putative target mRNAs whose expression is inversely correlated to that of miR-145-5p and that could participate to the maintenance of TET phenotype. Moreover, starting from the analysis of mRNAs differently expressed between tumoral (T) and normal (N) samples we identified several genes deregulated in TET and belonging to different signaling pathways related, for example, to cell adhesion and motility pathways or to cancer phenotype such as MAPK and Jak-Stat signaling pathways. Interestingly, the pathways that resulted significantly enriched in tumor vs normal tissues include signaling networks related to immune-related functions (as for example B cell receptor signaling, Chemokine signaling, Cytokine-cytokine receptor pathways). In particular we noticed that the majority of genes belonging to B cell receptor signaling (16 out of 19), to Chemokine signaling (23 out of 29) or to Cytokine-cytokine receptor pathways (33 out of 37) were down-regulated in tumoral vs normal tissues indicating a loss of the immune-related functions in TET.

A deeper characterization of deregulated genes and miRNAs in TET could lead to the identification of relevant players involved in the physiopathological function of thymic epithelial tissue.

## Conclusion

In summary these results suggest that the epigenetic regulation of the miR-145-5p expression, as well as the modulation of its functional targets, could be a relevant player in tumor progression and treatment response in TET. Further study in a larger thymic tumor series will allow the identification of miR-145-5p target genes specifically associated to each subtype and the relevance of the identified genes for malignancy progression and treatment response in TET.

## Additional files

Additional file 1: Primers used for RT-qPCR and ChIP. (DOCX 18 kb)

Additional file 2: RNAs deregulated in 6 Tumor (T) versus 3 Normal (N) samples. (XLSX 256 kb)

Additional file 3: A-C) Principal Component Analysis (PCA) of the gene expression data representing the distribution of samples. Each dot represents a sample and each color represents the type of the sample. (PDF 232 kb)

Additional file 4: Enriched pathways analysis of deregulated genes in Tumor versus Normal samples by David tools (*p*-value<0,05). The relative Pie-Chart is shown in Fig. 1d. (XLSX 9 kb)

Additional file 5: Putative targets of the best 9 of 22 miRNAs common to histotype groups of thymic tumors compared to normal tissue (Ganci et al., [43]) selected by target prediction analysis of genes deregulated in Thymoma versus normal Thymus tissues. (XLSX 179 kb)

Additional file 6: Enriched pathways analysis of the deregulated genes in Tumor versus Normal samples predicted to be the putative targets of t 9 miRs (see Ganci et al., [43]) by David tools (*p*-value<0,05). The relative Pie-Chart is shown in Fig. 1f. (XLSX 8 kb)

Additional file 7: Putative targets of miR-145-5p. (XLSX 29 kb)

Additional file 8: A) Luciferase reporter assay using vectors encoding the partial sequence of the 3’- UTR of Golm-1 mRNA (WT) and the mutant derivative lacking the putative miR-145-5p recognition sequences (position 260-267 of the Golm-11 3’-UTR) (DEL) were co-tansfected for 48h with 20 nM of miR-145-5p or negative control (SCR) in TC1889 cells. Renilla luciferase values were normalized to firefly luciferase values. B) Luciferase reporter assay using the vector encoding the 3’-UTR of CDH2 mRNA carrying a putative miR-145-5p binding site (pmirGLO-3’-UTR ) or the empty vector (pmirGLO) that were co-transfected for 48h with 5 nM miR-145-5p or negative control (SCR) in TC1889 cells. Firefly luciferase activity was normalized to Renilla luciferase expression for each sample. C) RT-qPCR to evaluate the expression levels of miR-145-5p after the treatment of TC1889 cells with the pre-miRNA145 (145-5p) and pre-miRNA Precursor Negative Control (SCR). D) Evaluation of the impact siRNA for CDH2 (siCDH2) in TC1889 by migration assay after 72h; E) evaluation by RT-qPCR of miR-145-5p expression after the treatment of TC1889 cells with the inhibitor of miR-145-5p (INH 145) or negative control (INH C). *P*-value was calculated by unpaired t-test and a value of P.0.05(*) and P.0.01(**) was considered statistically significant. (PDF 772 kb)

## Abbreviations

CDDP: Cisplatin
CDH2: N-cadherin
ChIP: Chromatin Immunoprecipitation
CK19: Cytokeratin 19
DsiRNAs: Dicer-substrate short interfering RNAs
EGF: Epithelial growth factor
EGFR: Epidermal growth factor receptor
FFPE: Formalin-fixed paraffin embedded
Golm1: Golgi membrane protein 1
HDACs: Histone deacetylases
IHC: Immunohistochemistry
INH-145: miR-145-5p inhibitor
INH-C: Scramble inhibitor
miRNAs: microRNAs
MPM: Malignant pleural mesothelioma
PCA: Principal component analysis
TC: Thymic carcinoma
TET: Tumor epithelial tissues
VPA: Valproic acid
WHO: World Health Organization
