# Supplementary material for: Thymic Epithelial Tumors phenotype relies on miR-145-5p epigenetic regulation
Source: Mol Cancer. 2017 May 10;16:88. doi: 10.1186/s12943-017-0655-2 (PMC5424390; doi:10.1186/s12943-017-0655-2)
Supplement: Supplementary file 1 — Primers used for RT-qPCR and ChIP. (DOCX 18 kb) [file 12943_2017_655_MOESM1_ESM.docx]

| Primers used for RT-qPCR and ChIP | |
| --- | --- |
| Gene | **Sequence** |
| GOLM1 fw | 5’-CAGCGTGAAAAGCGGAATC-3’ |
| GOLM1 rw | 5’-TCGGCCCTGTTGTGAAATA-3’ |
| PSAT1 fw | 5’-CCTCGGTCCTGGAATACAAG-3’ |
| PSAT1 rw | 5’-GCCAGCTTCTGAACGTCTTC-3’ |
| CDH2 fw | 5’-TGGACCGAGAATCACCAAATGT-3’ |
| CDH2 rw | 5’-ACACTTGAGGGGCATTGTCA-3’ |
| GAPDH fw | 5’-GAGTCAACGGATTTGTCGT-3’ |
| GAPDH rw | 5’-GACAAGCTTCCCGTTCTCAG-3’ |
| RPL19 fw | 5’-ATGACTGCATCGTTGATAAAATCC-3’ |
| RPL19 rw | 5’-GGCGCAAAATCCTCATTCTC-3’ |
| PCDH9 fw | 5’-GCATATTGTCACTTAGGTCAAACCA-3’ |
| PCDH9 rw | 5-GTCATGCCTTAACAAAAACCTCCT-3’ |
| EGFR fw | 5’-TCCCTCAGCCACCCATATGT-3’ |
| EGFR rw | 5’-AATGACAAGGTAGCGTGGG-3’ |
| CLDN1 fw | 5’-CCCTATGACCCCAGTCAATG-3’ |
| CLDN1 rw | 5’-GTTTTGGATAGGGCCTTGGT-3’ |
| H3 fw | 5’-GTGAAGAAACCTCATCGTTACAGGCCTGGT-3’ |
| H3 rw | 5’-CTGCAAAGCACCAATAGCTGCACTCTGGAA-3’ |
| ChIP R1 fw | 5’-CTTGTGATGCTGGGGAAGTT-3’ |
| ChIP R1 rw | 5’-GGGCTCAGAAAGAGAAAGCA-3’ |
| ChIP R2 fw | 5’-CAACAATGAGCCCTGGAAAG-3’ |
| ChIP R2 rw | 5’-CCGGAGCCAAGGTTAGAAGT-3’ |
| ChIP cyclin B1 intron fw | 5’- GGAGTCTCTATCGGCTCTTATACCG-3’ |
| ChIP cyclin B1 intron rw | 5’- GTCCAGTTTCCCAAGGCCAAT-3’ |
