# Supplementary material for: Thymic Epithelial Tumors phenotype relies on miR-145-5p epigenetic regulation
Source: Mol Cancer. 2017 May 10;16:88. doi: 10.1186/s12943-017-0655-2 (PMC5424390; doi:10.1186/s12943-017-0655-2)
Supplement: Supplementary file 3 — A-C) Principal Component Analysis (PCA) of the gene expression data representing the distribution of samples. Each dot represents a sample and each color represents the type of the sample. (PDF 232 kb) [file 12943_2017_655_MOESM3_ESM.pdf]

Figure S1

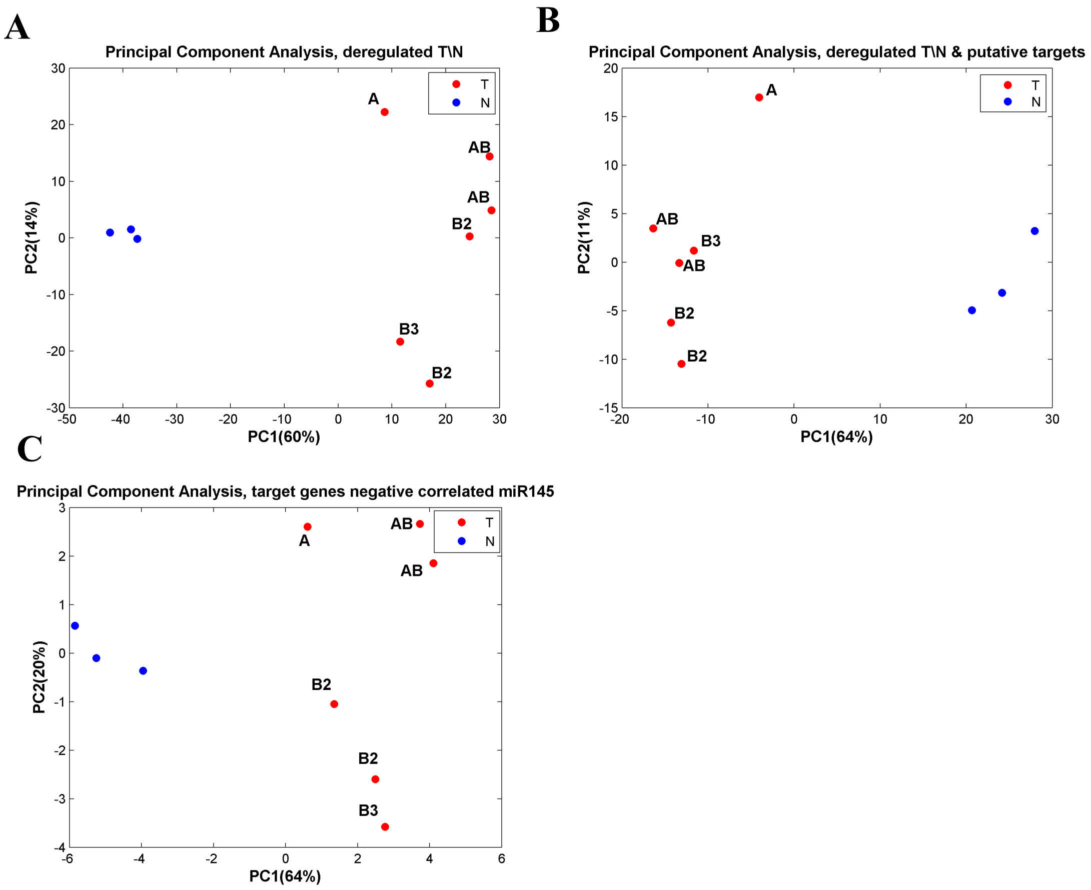

Figure Legend: A-C) Principal Component Analysis (PCA) of the gene expression data representing the distribution of samples. Each dot represents a sample and each color represents the type of the sample.
