# Supplementary material for: Thymic Epithelial Tumors phenotype relies on miR-145-5p epigenetic regulation
Source: Mol Cancer. 2017 May 10;16:88. doi: 10.1186/s12943-017-0655-2 (PMC5424390; doi:10.1186/s12943-017-0655-2)
Supplement: Supplementary file 8 — A) Luciferase reporter assay using vectors encoding the partial sequence of the 3’- UTR of Golm-1 mRNA (WT) and the mutant derivative lacking the putative miR-145-5p recognition sequences (position 260-267 of the Golm-11 3’-UTR) (DEL) were co-tansfected for 48h with 20 nM of miR-145-5p or negative control (SCR) in TC1889 cells. Renilla luciferase values were normalized to firefly luciferase values. B) Luciferase reporter assay using the vector encoding the 3’-UTR of CDH2 mRNA carrying a putative miR-145-5p binding site (pmirGLO-3’-UTR ) or the empty vector (pmirGLO) that were co-transfected for 48h with 5 nM miR-145-5p or negative control (SCR) in TC1889 cells. Firefly luciferase activity was normalized to Renilla luciferase expression for each sample. C) RT-qPCR to evaluate the expression levels of miR-145-5p after the treatment of TC1889 cells with the pre-miRNA145 (145-5p) and pre-miRNA Precursor Negative Control (SCR). D) Evaluation of the impact siRNA for CDH2 (siCDH2) in TC1889 by migration assay after 72h; E) evaluation by RT-qPCR of miR-145-5p expression after the treatment of TC1889 cells with the inhibitor of miR-145-5p (INH 145) or negative control (INH C). P-value was calculated by unpaired t-test and a value of P.0.05(*) and P.0.01(**) was considered statistically significant. (PDF 772 kb) [file 12943_2017_655_MOESM8_ESM.pdf]

Figure S2

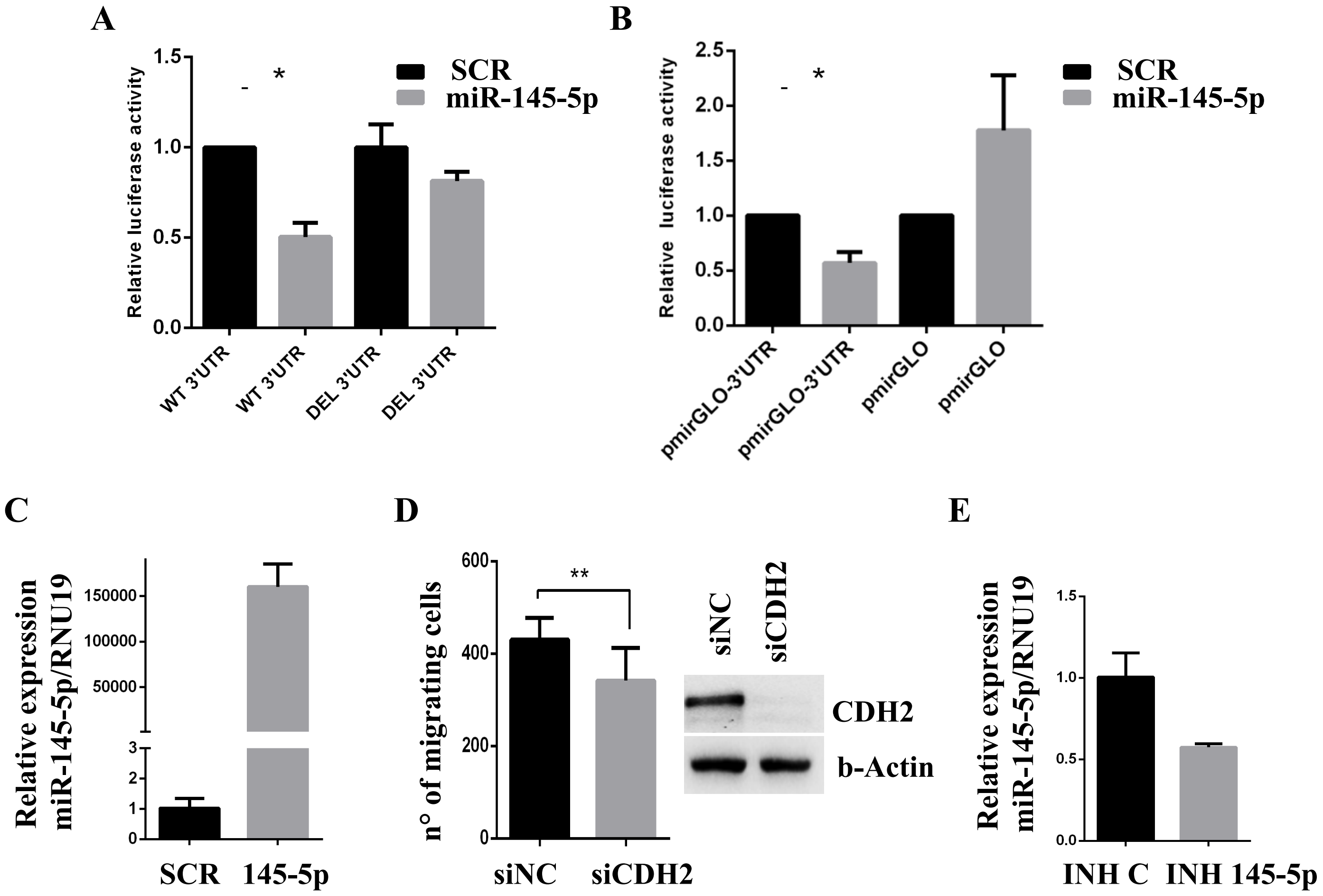

Figure Legend: A) Luciferase reporter assay using vectors encoding the partial sequence of the 3'-UTR of Golm-1 mRNA (WT) and the mutant derivative lacking the putative miR-145-5p recognition sequences (position 260-267 of the Golm-11 3'-UTR) (DEL) were co-tansfected for 48h with 20 nM of miR-145-5p or negative control (SCR) in TC1889 cells. Renilla luciferase values were normalized to firefly luciferase values. **B)** Luciferase reporter assay using the vector encoding the 3'-UTR of CDH2 mRNA carrying a putative miR-145-5p binding site (pmirGLO-3'-UTR) or the empty vector (pmirGLO) that were co-transfected for 48h with 5 nM miR-145-5p or negative control (SCR) in TC1889 cells. Firefly luciferase activity was normalized to Renilla luciferase expression for each sample. **C)** RT-qPCR to evaluate the expression levels of miR-145-5p after the treatment of TC1889 cells with the pre-miRNA145 (145-5p) and pre-miRNA Precursor Negative Control (SCR). **D**) Evaluation of the impact siRNA for CDH2 (siCDH2) in TC1889 by migration assay after 72h; **E)** evaluation by RT-qPCR of miR-145-5p expression after the treatment of TC1889 cells with the inhibitor of miR-145-5p (INH 145) or negative control (INH C).

P-value was calculated by unpaired t-test and a value of P≤0.05(*) and P≤0.01(**) was considered statistically significant.
